# Indices of human impacts on landscapes: How do they reflect the proportions of natural habitats?

**DOI:** 10.1515/biol-2025-1085

**Published:** 2025-04-28

**Authors:** Markéta Šantrůčková, Katarína Demková, Tomáš Frantík, Jiří Dostálek

**Affiliations:** Silva Tarouca Research Institute for Landscape and Ornamental Gardening, Květnové náměstí 391, CZ-252 43 Průhonice, Czech Republic; Institute of Botany, Academy of Sciences of the Czech Republic, CZ-252 43 Průhonice, Czech Republic

**Keywords:** human impact, indices, natural habitats, CORINE land cover, Czech Republic

## Abstract

Human activities significantly influence landscapes, altering natural habitats and ecosystem services. This study examines the relationship between human impacts, measured by the hemeroby index and coefficient of anthropogenic impact (CAI´), and the presence of natural habitats in the Czech Republic. Using CORINE land cover data and natural habitat mapping, we analysed national and regional scales to assess the effectiveness of these indicators in reflecting environmental changes. Compared with the simple anthropogenic impact coefficient (CAI´), the hemeroby index, which accounts for both the quantity and quality of ecosystems, provides more detailed insights. At the national level, both indices had an equally close relationship with the proportion of natural habitats, but at the regional level, the results for the hemeroby index were better. Our findings indicate a strong negative correlation between human impacts and the proportion of natural habitats, emphasizing the importance of refined indicators for environmental monitoring and policy-making. The advantage of both indices is that they could be easily calculated from satellite images and/or land cover data. Therefore, they could be used worldwide.

## 1 Introduction

Human activities have become a dominant factor leading to marked changes in most cultivated landscapes on Earth [1]. Ample evidence of the sheer scale of unprecedented human activities on Earth has been documented by many authors [2]. This implies that landscapes can be seen as contingent and historically variable outcomes of an interplay between socioeconomic and biophysical forces [3]. Research on the interactions between human activities and landscapes, especially with respect to ecosystem services and the contribution of ecosystems to human wellbeing [4], is often utilized to inform policy and decisions in various contexts, such as biodiversity conservation, natural resource management, and spatial planning [5,6,7]. Decision-makers are increasingly interested in such assessments [8,9].

Therefore, measuring the intensity of changes in land use is one of the key issues in the assessment of anthropogenic impacts [10]. In addition to the regular recording of land use data, an evaluation method and appropriate indicators are needed [11]. The intensity of land use can be approached from different content and structural aspects [12].

A wide range of criteria for developing (i.e., selecting and generating) ecosystem-service and ecological quality indicators on the basis of the literature and practical experience has been identified by several authors [13,14,15,16]. Ecological landscape indicators have also been integrated into methodologies for biodiversity indicators [17,18,19]. Despite the fact that many ecological landscape indicators have been applied thus far with efforts to select indicators and organize them according to the widely used categories of credibility, salience and legitimacy related to feasibility [13,15], their practical application is in many cases still problematic, mainly due to resource and technical limitations [20].

One of the indicators of human impacts on biodiversity is the natural capital index. It has two basic components: ecosystem quantity and ecosystem quality [21]. It was originally proposed for widespread international application; however, Czúcz et al. [18] modified the index for a low-level policy context using fine-scaled data.

Sowińska-Świerkosz [15] proposed an indicator of ecological landscape quality that enables the capture of different landscape characteristics treated as crucial for overall ecological quality. The indicator is composed of three variables: first, the normalized Shannon diversity index modified by the weighting score, which takes into account the degree of ecological significance of different land cover forms; second, the index is associated with the impact on the ecological quality of river valleys, which is calculated as a ratio of the length of rivers with curved shapes to the total area; and third, the index considers the proportion of ecological barriers (roads, railways) as a significant factor that decreases the ecological quality.

The degree of landscape fragmentation published by Jäger et al. [22] is also considered a suitable indicator of landscape quality. The process of splitting habitats into smaller isolated patches by urbanization and transport networks endangers and results in the loss of species (biodiversity). The degree of landscape fragmentation has been computed, e.g., for Switzerland [22], for the Czech Republic [23], and for the European Natura 2,000 site network [24,25].

For analysis of the human impacts of land use changes on landscapes, the concept of hemeroby was suggested as an indicator of naturalness for the European Union [26,27]. It enables an assessment and temporal comparison of landscapes in which relative changes over time are more important than absolute values [28].

Indicator of hemeroby has been applied to smaller urbanized areas [29,30] or regions [31] and at the level of entire countries. A map of hemeroby for Austria in relation to distance to nature and biodiversity was created [3,28,32]. The concept of hemeroby indicators for landscape monitoring has also been applied throughout Germany [11], Austria [33], Finland [34], Hungary [35], and Lithuania [36]. Hemeroby index was applied in the context of urban parks in Korea [37] or wetland ecosystems [38] and resource-based cities in China [39].

In the Czech Republic, the coefficient of anthropogenic impact on landscapes and the coefficient of ecological stability are commonly used to assess anthropogenic pressure [40,41,42]. The coefficient of anthropogenic impact on landscapes (CAI) represents the ratio of areas intensively used (arable land, built-up areas, and other areas) to areas less intensively used – with lower anthropogenic pressure (forestland, pastures, meadows, and water areas) in a given territorial unit [43,44]. The coefficient of ecological stability expresses the proportion of relatively stable (forests, pastures, meadows, and water bodies) to relatively unstable (arable land, built-up areas, and other areas) areas [45].

The aim of this study was to assess, to what extent, the common indicators of anthropogenic impacts (hemeroby and CAI indices) in accordance with the presence of natural habitats, which have important functions for ecosystem services and biodiversity conservation. For this purpose, we used indicators of hemeroby and CAI that are easy to calculate on the basis of the GIS analysis published by Walz and Stein [11] and Kupková [43]. Using CORINE land cover data and natural habitat mapping data for the Czech Republic, we aim (i) to verify the application of selected indicators to small regions and the entire country and (ii) to evaluate the relationships of the hemeroby and CAI indices with the proportion of the total area of natural habitats or with the proportions of their individual types.

## 2 Study area

**Figure 1:**
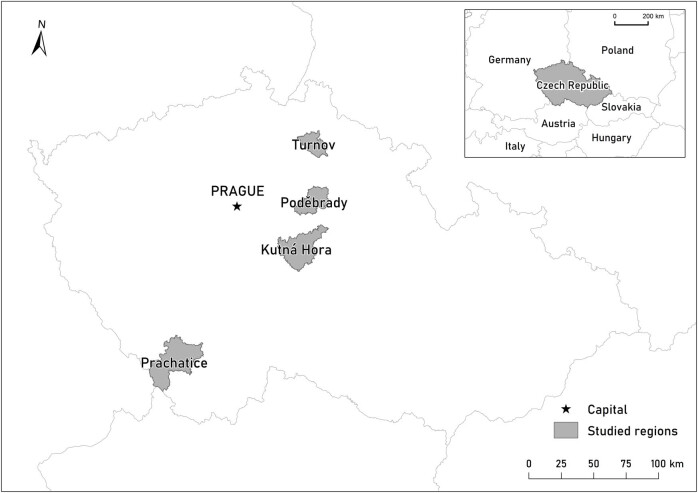
Study area.

Data were analysed at the national level (the Czech Republic) and the regional level because we compare the applicability of the indices on different scales (Figure 1). The delineation of the model areas respected the administrative boundaries of the state and middle-scale administrative units (regions). The main criteria for the selection of the four regions for detailed analysis were the altitude and land cover. The selected region represents lowlands (Poděbrady), highlands (Kutná Hora and Turnov), and mountains (Prachatice). The gradient from the most man-influenced land cover (Poděbrady) to the less man-influenced (Prachatice) was followed. The regions have different natural conditions what was also important for the selection. Last but not least, two regions are significantly covered by the natural protected areas (Turnov, Prachatice) and two are without large-scale natural protection (Poděbrady, Kutná Hora).

The Czech Republic is a hilly country in Central Europe with lowlands in the central and southeastern parts. The highlands are situated in the border region. The highest point is at an altitude of 1,602 m a.s.l. The territory has a moderate climate with an average annual temperature of approximately 7.5°C and precipitation of 674 mm. It is drained by a dense river network. The majority of the country is situated within a zone of broad-leaved deciduous forest, whereas the southeastern part borders the forest–steppe zone. Current vegetation cover is a result of abiotic conditions, biogeographical processes, and human activities; forest covers 33.9% of the country, agricultural land covers 53.3%, water area covers 2.1%, and built-up area covers 1.7% [46].

The Turnov region (with an area of 247 km^2^) in the northern part of the Czech Republic is characterized by hilly terrain with deep valleys (the highest point at an altitude of 744 m a.s.l.). Sand rock “towns” (groups of rocks) are typical attributes of the relief in the northeastern and southern parts of the model area. These parts are involved in the protected landscape area called the Bohemian Paradise, which has traditional architecture and high-levels of tourism. The western part of the model area has an agricultural‒forest landscape. Forests are the prevalent land cover type.

The Kutná Hora region (643 km^2^) is located in the central part of the Czech Republic and features hilly terrain (the highest point is 555 m a.s.l.) between the Elbe and Sázava Rivers. Only the northeastern part borders the flat terrain with an elevation of approximately 200 m a.s.l. The landscape of the model area has mainly agricultural–forest characteristics with scattered vegetation, small streams, remaining urbanism, and traditional architecture.

The Poděbrady region (348 km^2^) is characterized by flat terrain (the highest point is 299 m a.s.l.). It has predominantly agricultural characteristics, with tourism concentrated in the spa town of Poděbrady and its surroundings.

The Prachatice region (841 km^2^) is situated in the southern Czech Republic, where the Šumava Highlands (highest peak is 1,378 m a.s.l.) cover almost half of the model area. The northern part has hilly characteristics at altitudes ranging from 400 to 1,000 m. Forests cover the majority of the Prachatice region, and only the northern part has agricultural characteristics. The southern part is protected as the Šumava national park and the protected landscape area. On the eastern Prachatice region borders the Blanský les (forest) protected landscape area. The region is typical with a landscape structure, with small landscape features such as linear greenery and high-levels of tourism remaining in the highlands.

## 3 Data and methods

### 3.1 Data sources

*CORINE Land Cover 2018* (CLC) (European Environmental Agency) is a vector database classified and digitized on the basis of satellite images with 100 m positional accuracy and a 25 ha minimum mapping unit using the standardized CLC nomenclature (44 CLC classes). In the Czech Republic, 29 CLC classes were identified at a scale of 1:100,000. The data were used to quantify the level of human impacts on the landscape.

**Figure 2:**
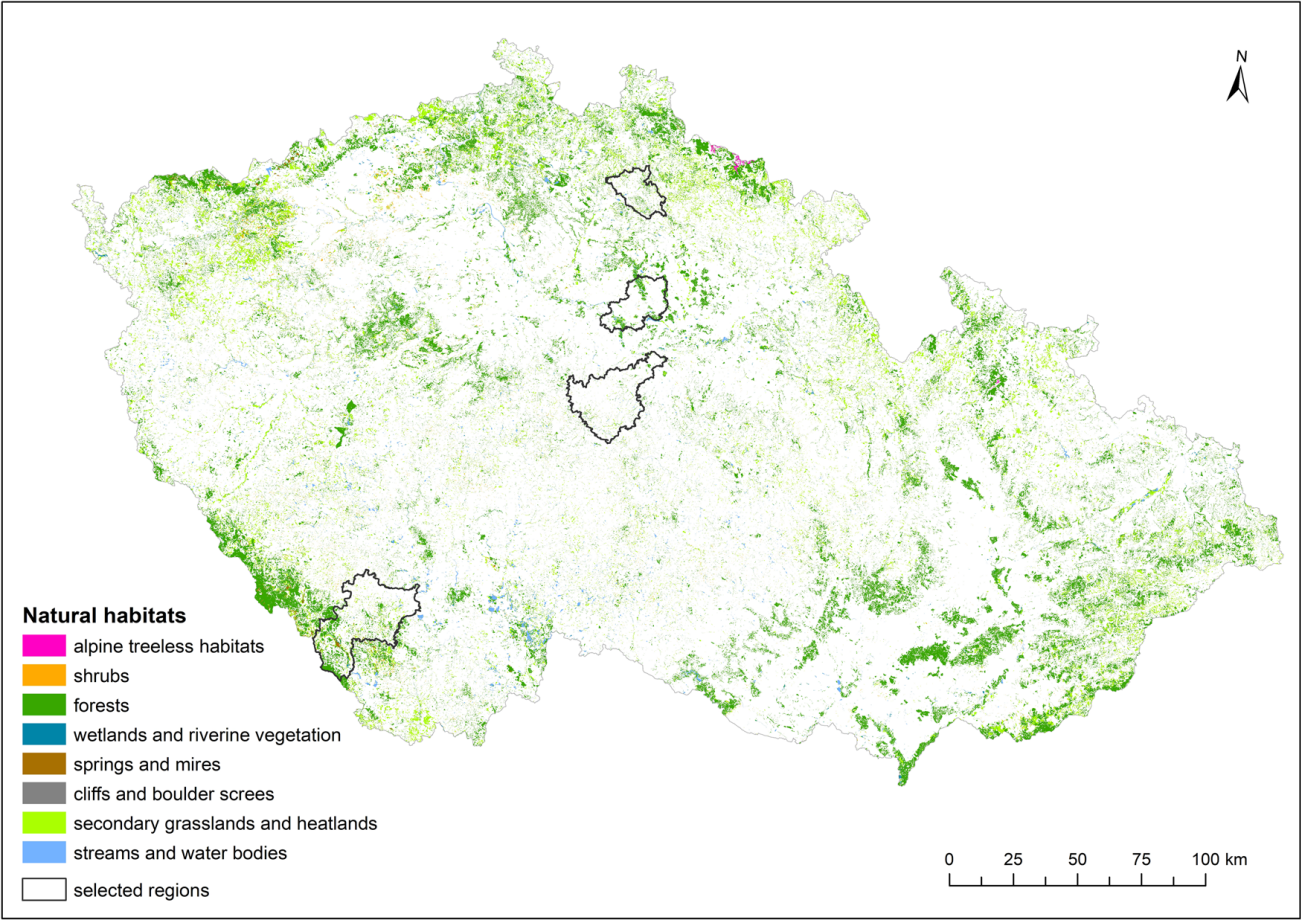
Distribution of basic groups of natural habitats in the Czech Republic. Source: The Nature Conservation Agency of the Czech Republic.

*Natural habitat mapping data* (the Nature Conservation Agency of the Czech Republic, ©AOPK) are vector data for the entire territory of the Czech Republic at a scale of 1:10,000 and were developed during the establishment of NATURA 2000. This dataset is continuously updated. The version from November 2020 was used for analysis. The following basic groups of natural habitats according to Chytrý et al. [47] were used in our analysis: streams and water bodies, wetlands and riverine vegetation, springs and mires, cliffs and boulder screes, secondary grasslands and heathlands, and shrubs and forests (for their proportions in the studied regions, see Table 1; for distribution, see Figure 2).

**Table 1:** Area and proportion of basic groups of natural habitats in the studied regions

Area	Scrubs	Forests	Wetlands and riverine vegetation	Springs and mires	Cliffs and boulder screes	Secondary grasslands and heathlands	Streams and water bodies	Natural habitats in total
**Czech Republic**								
ha	23603.94	621031.88	10601.16	5844.00	1027.92	249008.91	35436.47	947471.57
%	0.30	8.00	0.14	0.08	0.01	3.21	0.46	12.21
**Kutná Hora Region**								
ha	88.96	1121.69	59.09	0.17	4.14	672.65	203.81	2150.50
%	0.17	2.13	0.11	0.00	0.01	1.28	0.39	4.08
**Poděbrady Region**								
ha	52.52	2809.78	121.09	5.34	0	418.96	264.51	3672.21
%	0.19	9.96	0.43	0.02	0	1.49	0.94	13.02
**Prachatice Region**								
ha	284.71	9587.23	239.33	683.95	9.98	3167.62	247.39	14223.18
%	0.39	13.28	0.33	0.95	0.01	4.39	0.34	19.70
**Turnov Region**								
ha	20.15	1429.46	27.30	0.99	13.77	575.40	86.92	2153.98
%	0.11	7.81	0.15	0.01	0.08	3.14	0.47	11.77

*The potential natural vegetation map* [48] was prepared at the national level at a scale of 1:500,000 and expresses the extent of natural vegetation without any human impacts on ecosystems.

### 3.2 Data processing

First, a regular grid network of 1 km^2^ was created. An advantage of grid maps is their spatial and temporal comparability, which is the opposite of using administrative units [49]. Cells on the state border that reach partially outside the border were excluded. Only cells with 100% coverage from the Czech Republic were used in the analysis. The total number of grid cells included in the analysis was 77,615. The proportions of natural habitat classes in each grid cell were calculated. To distinguish intensively used landscapes from natural landscapes, the coefficient of anthropogenic impact on landscapes [43,44] and the index of hemeroby [11] were computed in each grid cell. CORINE land cover data (classes at the third level) were used for calculating both indices.

The coefficient of anthropogenic impact on landscapes is a simple division (share) of artificial and highly intensively used surfaces (*A*), such as urban spaces, mine sites, sport and leisure facilities, intensive agricultural areas and crop lands (arable land, vineyards, gardens, orchards, etc.), and areas less intensively used and natural or seminatural areas (*N* – forest, shrubs, grasslands, wetlands, and water bodies):

\[\text{CAI}=A\times {N}^{-1}]\]

In rare cases where the grid cell included less than 0.0001% (i.e., 1 m^2^) of less intensively used areas, the CAI value was set at 1,000,000. The CAI index was then transformed to CAI′: CAI′ = LOG (CAI + 1), which adjusted the values from 0 to 6. This index was used as a measure of human impact on landscapes, where 0 indicates natural/lowest impact, and 6 indicates artificial/highest impact. However, it does not reflect the quality of ecosystems. For example, there is no difference between natural-leaf forest and planted spruce monoculture. Both forests are always considered natural.

In an effort to involve the quality of ecosystems in the computation, the index of hemeroby was used. Walz and Stein [11] distinguished CLC classes according to 7 degrees of hemeroby (Table 2). The assignment to a special degree of hemeroby reflects the intensity, duration, and range of human impact [50]. While residential areas are characterized by a high degree of anthropogenic impact on ecosystems, which are mostly long in duration, agricultural and forest lands have different intensities of use. Thus, an additional intersection with potential natural vegetation [48] was necessary. Therefore, forests were classified by the extent of their deviation from potential natural vegetation [11]. This step was performed only for classes such as deciduous, coniferous, and mixed forests. For example, a montane spruce forest at high altitudes, which is covered by a natural coniferous forest typical for such conditions, was assigned a lower hemeroby degree than nonnative spruce monocultures in the lowlands where deciduous forests should grow. Currently, there is no area without any human impact in Central Europe [51]. Every ecosystem has been affected by pollution emissions or climate change; therefore, only bare rocks were assigned to the lowest degree of hemeroby. The index of hemeroby was computed as a simple area-weighted hemeroby index [11]:

\[M=\mathop{\sum }\limits_{h=1}^{n}{f}_{n}\times h,]\]

where *n* is the number of degrees of hemeroby (*n* = 7), *f*_*n*_ is the proportion of category *n*, and *h* is the degree of hemeroby.

**Table 2:** Assignment of the degree of hemeroby to CORINE land cover (CLC) classes [11]

Degree of hemeroby	CLC class
1. Ahemerobic – almost no human impact	332 Bare rocks
2. Oligohemerobic – weak human impact	311 Broad-leaved forest
	312 Coniferous forest (PNV*)
	313 Mixed forest (PNV*)
	411 Inland marshes
	412 Peat bogs
3. Mesohemerobic – moderate human impact	312 Coniferous forest (not PNV*)
	313 Mixed forest (not PNV*)
	321 Natural grasslands
	322 Moors and heathland
	324 Transitional woodland-shrub
	333 Sparsely vegetated areas
4. β-Euhemerobic – moderate-strong human impact	141 Green urban areas
	231 Pastures
	243 Land principally occupied by agriculture with significant areas of natural vegetation
	511 Water courses
	512 Water bodies
5. α-Euhemerobic – strong human impact	142 Sport and leisure facilities
	211 Non-irrigated arable land
	221 Vineyards
	222 Fruit trees and berry plantations
	242 Complex cultivation patterns
6. Polyhemerobic – very strong human impact	112 Discontinuous urban fabric
	131 Mineral extraction sites
	132 Dump sites
	133 Construction sites
7. Metahemerobic – excessively strong human impact, biocenosis destroyed	111 Continuous urban fabric
	121 Industrial or commercial units
	122 Road and rail networks and associated land
	123 Port areas
	124 Airports

*Potential natural vegetation [48].

## 4 Statistical evaluation

Obviously, the data obtained from the grid network are not independent values. Hence, the use of a simple correlation coefficient to test the relationship between indices of anthropogenic impact and the proportion of natural habitats is not possible. Therefore, we used two complementary statistical procedures at the level of the whole Czech Republic:

(1) Partial correlation – Correlation coefficients were controlled for the longitude and latitude of the grid cell. The output was *r*_part_ using Statistica v.12 software. The advantage of this approach is the use of the entire grid network. However, the procedure only partially considers the fact that the data are not independent.
(2) Permutation tests – The largest possible rectangle was selected from the whole grid network. Its size was 256 cells in the west‒east direction and 96 cells in the north‒south direction; therefore, out of a total of 77,615 grid cells, 24,576 grid cells were used for statistical analysis. The correlation coefficients were then calculated via the RDA permutation test in CANOCO software [52]. The permutation test was restricted to rectangular grids. The output was *r*_permut_. This procedure fully accounts for the fact that the data are not independent. However, the disadvantage is that only one-third of the grid network is used.

To increase the conclusiveness of the results at the national level, we considered only the relationships in which the correlation coefficient calculated by both the first and second procedures was statistically significant. At the regional level, only the first procedure (*r*_part_) was used because the regions could not be displayed as rectangles.

## 5 Results

### 5.1 Indices of human impact at the national level

**Figure 3:**
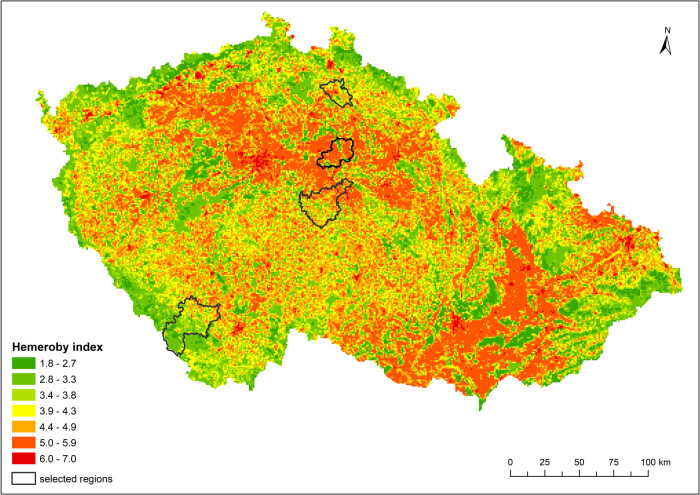
Distribution of the hemeroby index based on calculations for grid cells.

**Figure 4:**
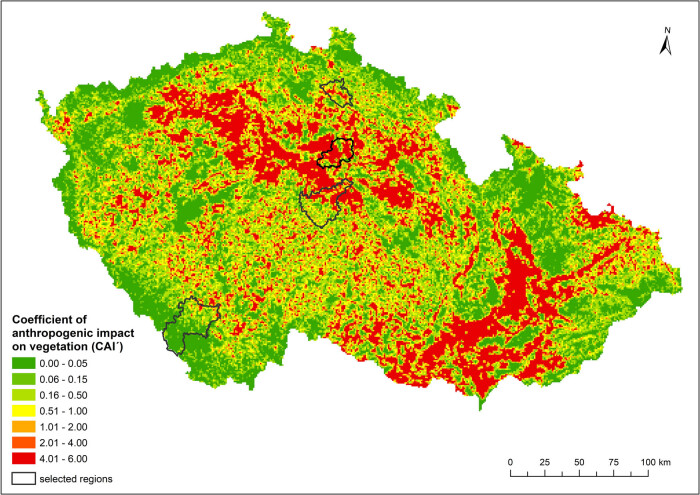
Distribution of the coefficient of anthropogenic impact on landscape (CAI´) based on calculations for grid cells.

The maps of human impacts on the landscape, which were calculated via two different indicators, and the distributions of natural habitats at the national level are shown in Figures 3 and 4.

From the maps, it is possible to observe certain differences, from which it follows that the use of the hemeroby index, which considers the proportion of individual degrees of anthropogenic impact in a given area, provides a more detailed view of the degree of human influence on the landscape than does the coefficient of anthropogenic impact (CAI), which is defined as the simple ratio of areas with a high intensity of use and areas with a lower intensity of use.

The overall relationships between the indices of anthropogenic impacts and the proportions of basic groups of natural habitats at the national level are presented in Table 3. The values of the hemeroby index were significantly negatively correlated with the proportions of most of the main groups of natural habitats; however, a significant relationship was not demonstrated for two groups of natural habitats (streams and water bodies, and wetlands and riverine vegetation). However, the values of CAI′ had a statistically significant negative correlation with natural biotopes in all cases (Table 3).

**Table 3:** Relationships between indicators of human impact (hemeroby index, CAI′) and the proportions of basic groups of natural habitats at the national level

		Scrubs	Forests	Wetlands and riverine vegetation	Springs and mires	Cliffs and boulder screes	Secondary grasslands and heatlands	Streams and water bodies	Natural habitats in total
**Czech Republic**									
Hemeroby index	*r*_part_	**−0.0277**	**−0.5762**	**−0.0071**	**−0.0986**	**−0.0510**	**−0.1433**	−0.0004	**−0.5474**
	*p*_part_	***p*** **= 0.000**	***p*** **< 0.001**	***p*** **= 0.047**	***p*** **< 0.001**	***p*** **< 0.001**	***p*** **< 0.001**	*p* = 0.920	***p*** **< 0.001**
	*r*_permut_	**−0.099325**	**−0.549309**	−0.018154	**−0.093544**	**−0.0908382**	**−0.185432**	**−0.025621**	**−0.515758**
	*p*_permut_	***p*** **< 0.002**	***p*** **< 0.002**	*p* = 0.182	***p*** **< 0.002**	***p*** **< 0.002**	***p*** **< 0.002**	***p*** **= 0.026**	***p*** **< 0.002**
CAI′	*r*_part_	**0.0309**	**−0.3041**	**−0.0245**	**−0.0390**	**−0.0271**	**−0.1857**	**−0.0468**	**−0.3445**
	*p*_part_	***p*** **< 0.001**	***p*** **< 0.001**	***p*** **< 0.001**	***p*** **< 0.001**	***p*** **< 0.001**	***p*** **< 0.001**	***p*** **< 0.001**	***p*** **< 0.001**
	*r*_permut_	**−0.0740**	**−0.3174**	**−0.0344**	**−0.0567**	**−0.0616**	**−0.2022**	**−0.0651**	**−0.3572**
	*p*_permut_	***p*** **= 0.018**	***p*** **< 0.002**	***p*** **= 0.020**	***p*** **= 0.004**	***p*** **< 0.002**	***p*** **< 0.002**	***p*** **< 0.002**	***p*** **< 0.002**

**Bold** indicates a significant correlation (*p* < 0.05).

The correlation coefficients (*r*_part and_ *r*_permut_) and probability level (*p*) are given.

### 5.2 Indices of human impact at the regional level

**Figure 5:**
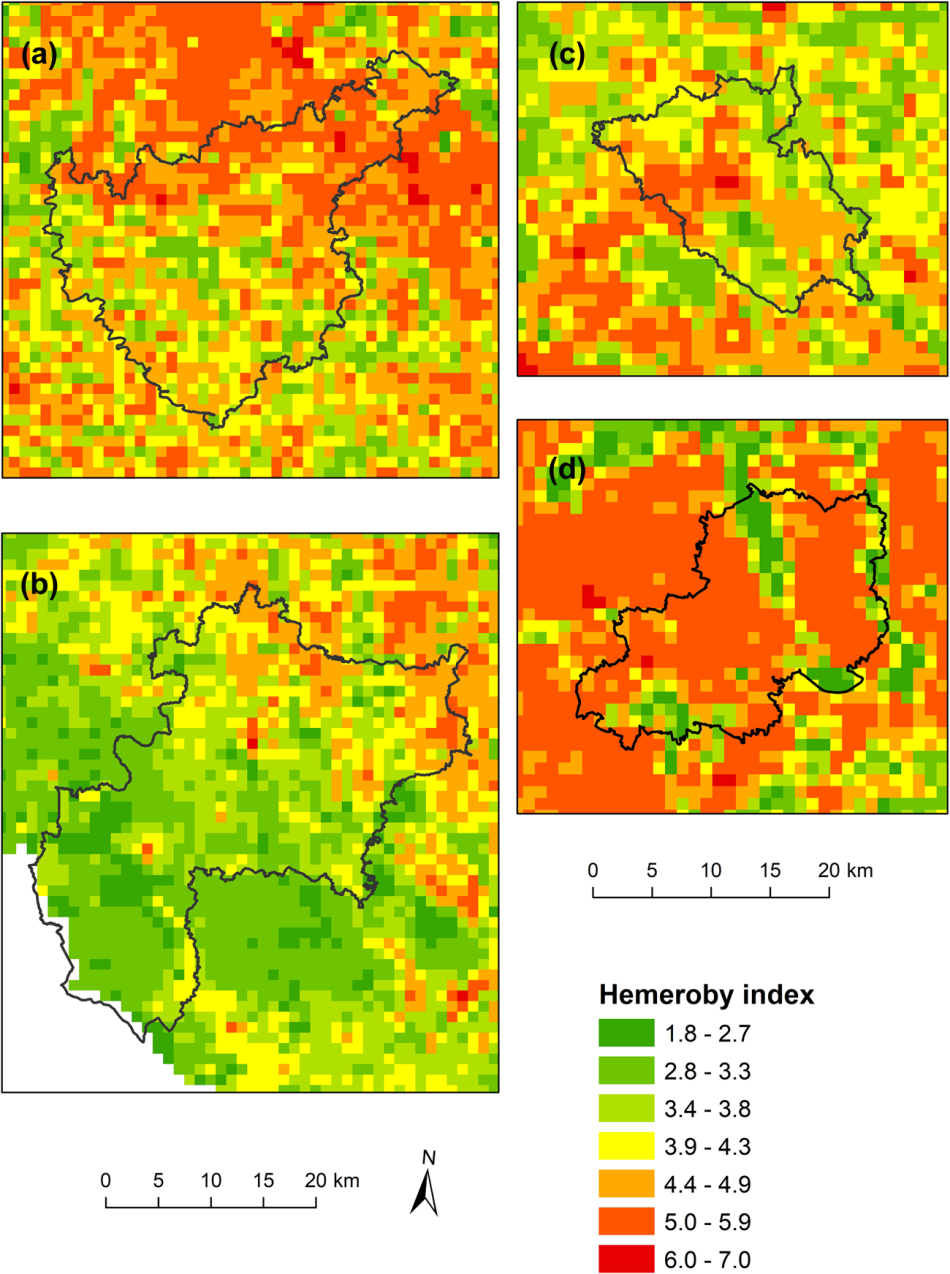
Distribution of the hemeroby index based on calculations for grid cells at the regional level (a – Kutná Hora, b – Prachatice, c – Turnov, d – Poděbrady).

**Figure 6:**
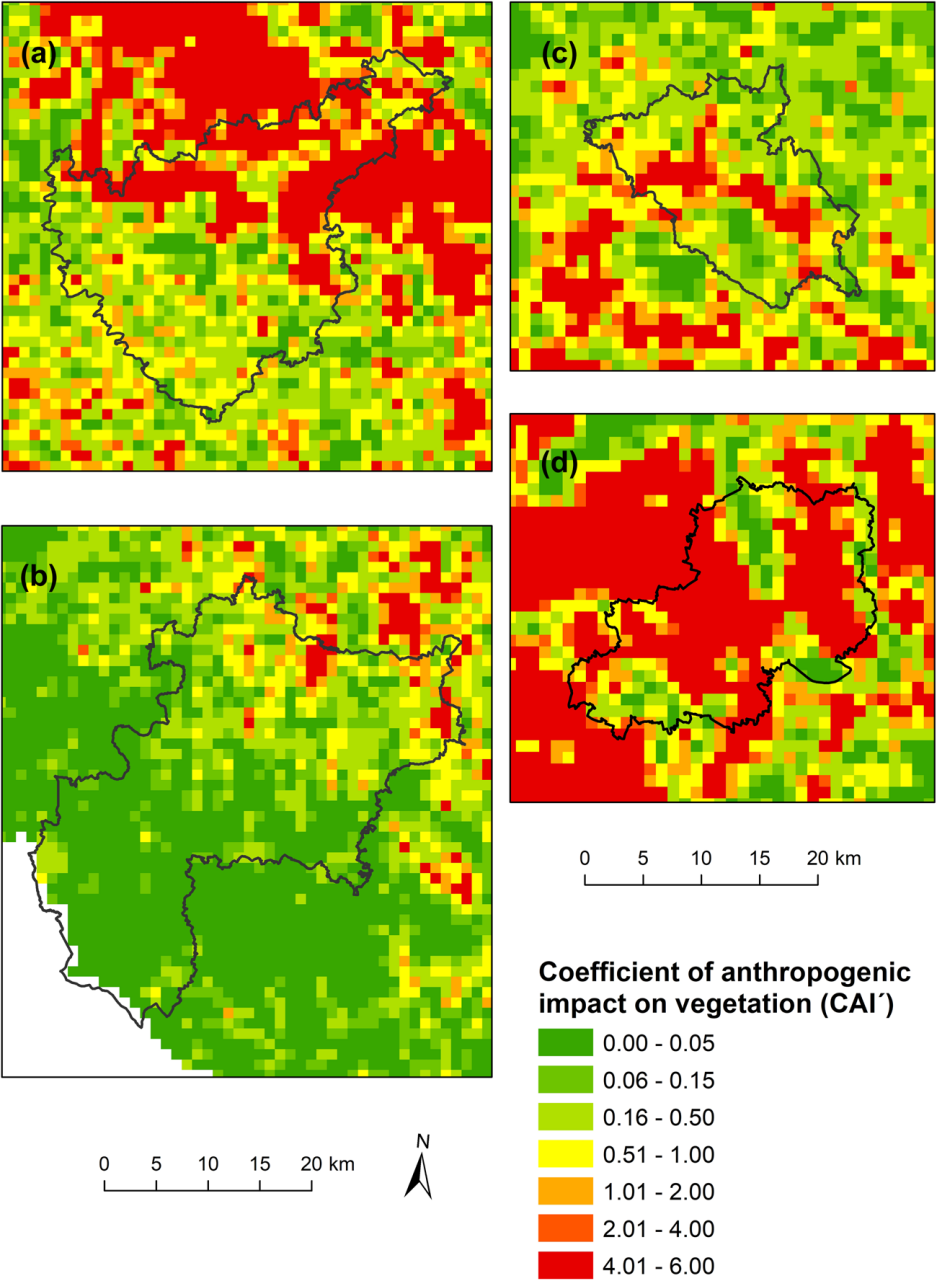
Distribution of the coefficient of anthropogenic impact on landscape (CAI´) based on calculations for grid cells at the regional level (a – Kutná Hora, b – Prachatice, c – Turnov, d – Poděbrady).

If the indices of human impact were used for the analysis of landscapes in small regions with different natural conditions (Figures 5 and 6), the relationships between the values of these indices and the proportion of natural habitats were in accordance with the results at the national level, which indicates that the greater the value of the anthropogenic impact on the landscape was, the lower the total representation of natural habitats (Table 4). The correlation between both indicators of human impact (hemeroby index, CAI′) and the proportion of natural habitats was understandably weaker because of much lower degrees of freedom but was still strong for natural habitats in total and in forests.

**Table 4:** Relationships between indicators of human impact (hemeroby index, CAI′) and the proportions of basic groups of natural habitats at the regional level

		Scrubs	Forests	Wetlands and riverine vegetation	Springs and mires	Cliffs and boulder screes	Secondary grasslands and heatlands	Streams and water bodies	Natural habitats in total
**Kutná Hora Region**									
Hemeroby index	*r*_part_	0.0256	**−0.2547**	−0.0166	−0.0510	**0.1026**	**0.1040**	**0.0866**	**−0.1210**
	*p*_part_	*p* = 0.558	***p*** **< 0.001**	*p* = 0.704	*p* = 0.244	***p*** **= 0.019**	***p*** **= 0.017**	***p*** **= 0.047**	***p*** **= 0.005**
CAI′	*r*_part_	0.0032	**−0.2694**	−0.0202	−0.0717	0.00110	0.0724	**0.0873**	**−0.1494**
	*p*_part_	*p* = 0.941	***p*** **< 0.001**	*p* = 0.645	*p* = 0.101	*p* = 0.802	*p* = 0.098	***p*** **= 0.046**	***p*** **= 0.001**
**Poděbrady Region**									
Hemeroby index	*r*_part_	−0.0035	**−0.9491**	**−0.3217**	**−0.2096**		**−0.1961**	**−0.2388**	**−0.9159**
	*p*_part_	*p* = 0.954	***p*** **< 0.001**	***p*** **< 0.001**	***p*** **< 0.001**		***p*** **= 0.001**	***p*** **< 0.001**	***p*** **< 0.001**
CAI′	*r*_part_	0.0136	**−0.6763**	**−0.3056**	**−0.1529**		**−0.2809**	**−0.2452**	**−0.6964**
	*p*_part_	*p* = 0.821	***p*** **< 0.001**	***p*** **< 0.001**	***p*** **= 0.010**		***p*** **< 0.001**	***p*** **< 0.001**	***p*** **< 0.001**
**Prachatice Region**									
Hemeroby index	*r*_part_	**−0.0748**	**−0.4366**	**−0.1013**	**−0.1593**	−0.0585	0.0511	−0.0115	**−0.4324**
	*p*_part_	***p*** **= 0.045**	***p*** **< 0.001**	***p*** **= 0.007**	***p*** **< 0.001**	*p* = 0.117	*p* = 0.171	*p* = 0.759	***p*** **< 0.001**
CAI′	*r*_part_	−0.0549	**−0.0732**	0.0038	0.0037	−0.0300	−0.0417	−0.0037	**−0.0844**
	*p*_part_	*p* = 0.141	***p*** **= 0.050**	*p* = 0.918	*p* = 0.921	*p* = 0.421	*p* = 0.263	*p* = 0.921	***p*** **= 0.024**
**Turnov Region**									
Hemeroby index	*r*_part_	−0.0327	**−0.7988**	−0.1424	**−0.1932**	**−0.3844**	**−0.2336**	−0.0021	**−0.7361**
	*p*_part_	*p* = 0.662	***p*** **< 0.001**	*p* = 0.056	***p*** **= 0.009**	***p*** **< 0.001**	***p*** **= 0.002**	*p* = 0.978	***p*** **< 0.001**
CAI′	*r*_part_	−0.0395	**−0.4563**	−0.0752	−0.1097	**−0.1931**	−0.2615	−0.0890	**−0.4707**
	*p*_part_	*p* = 0.597	***p*** **< 0.001**	*p* = 0.314	*p* = 0.141	***p*** **= 0.009**	***p*** **< 0.001**	*p* = 0.233	***p*** **< 0.001**

**Bold** indicates a significant correlation (*p* < 0.05).

The correlation coefficient (*r*_part_) and probability level (*p*) are given.

The significance of the correlation coefficients between the value of anthropogenic influence on the landscape and the representation of natural habitats in individual regions differed considerably both from the values at the national level and from each other.

In the Turnov and Poděbrady regions, a significant negative relationship between the degree of anthropogenic influence on the landscape and the proportion of grassland biotopes was demonstrated via both indices (hemeroby index, CAI′). On the other hand, in the Prachatice region, no relationship between anthropogenic impacts on the landscape and grassland biotope cover was found. An interesting paradox occurred in the case of the Kutná Hora region, where the area of grassland biotopes increased with increasing levels of anthropogenic impact, as expressed by the hemeroby index.

A significant relationship between the amount of scrubs and the degree of anthropogenic impact on the landscape was demonstrated only by the hemeroby index in the Prachatice region. In the other regions, the relationship between anthropogenic impact and the proportion of scrubs was not indicated.

The hemeroby index revealed that the occurrence of wetlands and riverine vegetation biotopes decreased significantly with increasing anthropogenic impact only in the Prachatice and Poděbrady regions. Using the CAI´ coefficient, the relationships between the presence of these biotopes and anthropogenic impacts were documented only in the Poděbrady region (Table 4).

Both indices (hemeroby index, CAI′) also revealed that the areas of streams and water bodies habitats decreased significantly with increasing anthropogenic impact only in the Poděbrady region. In the Kutná Hora region, on the other hand, the abundance of streams and water bodies biotopes increased with increasing anthropogenic impact. In the Turnov and Prachatice regions, the statistically significant relationship between the area of these biotopes and anthropogenic impact was not proven via the coefficients of human impact (hemeroby index, CAI´).

The loss of the area of springs and mires with increasing anthropogenic impact was statistically significantly correlated with the hemeroby index in all regions except Kutná Hora. With respect to the CAI´ indices, no significant relationship was detected between the anthropogenic impact and the area of this habitat group in all the regions except for Poděbrady.

Both the hemeroby index and CAI´ had a strong negative correlation between the proportion of cliffs and boulder screes and anthropogenic influence in the Turnov region, whereas in the Kutná Hora region, this correlation was weakly positive when only the hemeroby index was used.

## 6 Discussion

The overall results unsurprisingly confirmed a decrease in the proportion of natural habitats with increasing anthropogenic impact on the landscape, as expressed by the hemeroby and CAI´ indices. Similar findings have been documented by many previous studies [4,5,6,7,10].

CLC data have been widely used in different studies at different spatial scales. The spatial pattern of land cover (or land cover change) can provide direct measures of human activity [53]. Land use/land cover changes have been suggested as leading forces influencing biodiversity changes associated with habitat degradation or even loss [54] and have serious impacts on providing ecosystem services [55,56].

At the national level, the two indices of anthropogenic influence that were used (hemeroby index and CAI´) yielded the same results, but at the regional level, the hemeroby index was somewhat more closely related to the representation of natural habitats. The hemeroby index was proven to be more useful for studying human impacts on landscapes at the national level and especially at the regional level because of its construction. It reflects not only the quantity but also the environmental quality of different land cover types. In particular, forests are classified in more detail than in the CORINE land cover classification, which uses a forest classification based on the potential natural vegetation maps [48]. Similar forest classification was prepared for other Central European countries by Grabherr et al. [33] and Kowarik [57]. Grabherr et al. [33] proposed a detailed forest biotope classification and its relation to hemeroby for Austria. Kowarik [57] compared old approaches of forest classification and NATURA forest biotopes with the concept of hemeroby for Germany. Owing to the detailed land cover classification used for calculating the hemeroby index, it can be applied with good results for small spatial units [11,31,34,35]. Using these small spatial units, the hemeroby index was proven to be useful for large model areas as well as for small territorial units such as regions and municipalities.

The advantage of the coefficient of anthropogenic impact (CAI´) is its simple construction, which uses basic land cover data. However, the original CAI index was developed for general assessment of large territories [43,44] and is not suitable for small units. Extreme values can occur in such small units, or it is even not possible to count the index in a completely urbanized area without any natural surfaces. During the assessment of the anthropogenic impact on the landscape by CAI´ with respect to natural habitats, CAI´ had relatively good results, especially at the national level; however, it was still somewhat worse than the hemeroby index.

In terms of the presence of natural habitats at the national level, both in general and in terms of their most abundant basic groups of natural habitats, their decrease was attributed to increasing anthropogenic impacts on the landscape. The aforementioned statement was also true for three of the four studied regions – Poděbrady in the lowlands, Turnov in the highlands with sandy rocks, and Prachatice in the mountains. Kutná Hora, where the share of natural habitats in the region was the lowest and was usually small and dispersed, was somewhat different. The proportion of natural habitats as a whole was also relatively weakly negatively related to anthropogenic impacts on the landscape. However, this was not proven for secondary grasslands and heathlands, cliffs and boulder screes, and streams and water bodies. The explanation for this could be that natural habitats are small and isolated in deep valleys with steep slopes or in manor gardens [58,59]. Moreover, the prevailing land cover matrix consists of intensively used agricultural land or managed forests.

## 7 Conclusion

The indices of anthropogenic impact were very closely negatively correlated with the representation of natural habitats, especially forests, scrubs, and grasslands. For aquatic and wetland habitats, the relationships were relatively weak. Both indices (hemeroby index and CAI´) effectively reflected the proportion of natural habitats in the landscape and yielded almost the same results at the national level. We can assume that for a general assessment at the national level, the transformed coefficient of anthropogenic impact (CAI´), which is very easy to construct from only CORINE land cover data, yields sufficient results and could be used with good results. On the other hand, the hemeroby index usually has a closer relationship with the representation of natural habitats than CAI´ does at the regional level. Owing to the detailed land cover classification used for calculating the hemeroby index, it can be applied with better results for small spatial units. The advantage of both indices is that they could be easily calculated from satellite images and/or land cover data. Therefore, they could be used worldwide. In Europe, including the Czech Republic, this analysis could be used for an initial assessment of landscape ecological quality and the need for potential landscape restoration in accordance with the European Nature Restoration Law [60]. To conclude, the hemeroby index is a good solution for assessing anthropogenic impacts because of its relatively easy construction and reliable results at all spatial extents. The hemeroby index was proven to be useful for studying human impacts on landscapes at both the national and regional levels because it reflects not only the quantity but also the environmental quality of the land cover types.
